# Vaccination dropout and associated factors among children in Ethiopia: a systematic review and meta-analysis (2014–2024)

**DOI:** 10.1186/s12887-025-05786-3

**Published:** 2025-05-28

**Authors:** Eyasu Bamlaku Golla, Habtamu Geremew, Alegntaw Abate, Mohammed Ahmed Ali, Mulat Belay Simegn, Werkneh Melkie Tilahun, Samuel Abdisa Kuse, Smegnew Gichew Wondie

**Affiliations:** 1 College of Health Sciences, Oda Bultum University, Chiro, Ethiopia; 2 Department of Medical Laboratory Sciences, College of Health Science, Oda Bultum University, Chiro, Ethiopia; 3 Department of Midwifery, College of Health Sciences, Oda Bultum University, Chiro, Ethiopia; 4 Department of Public Health, College of Medicine and Health Sciences, Debre Markos University, Debre Markos, Ethiopia; 5 Department of Human Nutrition, College of Medicine and Health Sciences, Mizan Tepi University, MizanAman, Ethiopia

**Keywords:** Vaccine dropout, Associated factors, Ethiopia, Systematic review, Meta analysis

## Abstract

**Background:**

Children in sub-Saharan Africa, particularly in Ethiopia, continue to suffer from vaccine-preventable diseases (VPDs), contributing to child mortality. One of the key challenges is vaccination dropout, where children fail to receive subsequent doses after the initial vaccine, leading to incomplete immunization coverage. Hence, our review aimed to determine the pooled magnitude of vaccination dropout and the factors associated with it among children in Ethiopia.

**Method:**

A comprehensive search of relevant studies was conducted through electronic web-based international databases and the institutional repository of Ethiopian universities. Data were extracted via a Microsoft Excel spreadsheet and then exported to STATA 17 for statistical analysis. A checklist from the Joanna Briggs Institute was utilized to assess the quality of the studies. A random-effects model was employed to estimate the pooled magnitude of vaccination dropout. Heterogeneity between studies was evaluated via the I-square test. Funnel plots and Egger’s regression test were utilized to assess publication bias.

**Results:**

Seventeen articles with a total sample size of 9152 children (12–23 months old) were included in this meta-analysis. Consequently, the pooled magnitude of vaccination dropout from BCG to measles and Penta-1 to Penta-3 among children aged 12–23 months in Ethiopia was 16.93% (95% CI: 12.43, 21.44) and 13.16% (95% CI: 8.37, 17.96) respectively. Mothers who did not attend ANC (AOR = 3.58, 95% CI = 1.99, 6.44), postponed immunization schedule (AOR = 2.73, 95% CI = 1.53, 4.87), distance from the health care facility (AOR = 2.46, 95% CI = 2.01, 17.18), and home delivery (AOR = 2.78, 95% CI = 2.28, 3.38) were identified as significant factors associated with vaccine dropout.

**Conclusion:**

The overall pooled magnitude of vaccination dropout among children in Ethiopia is greater than the WHO recommendation of less than 10%. Therefore, our findings suggest the necessity of counseling and educating women to attend antenatal care (ANC) follow-ups, give birth at healthcare facilities, and provide mobile and outreach immunization services for remote areas.

**Supplementary Information:**

The online version contains supplementary material available at 10.1186/s12887-025-05786-3.

## Introduction

Vaccination is administering a vaccine to help the immune system develop immunity against a disease [1]. It is the most effective way to prevent infectious diseases and one of the most cost-effective public health measures [2]. The effectiveness of vaccination in preventing infectious diseases has been extensively researched and confirmed [3].

According to the World Health Organization (WHO), vaccination prevents 3.5–5 million child deaths annually [4]. Global vaccination coverage has experienced some recovery by 2022. However, 20.5 million children worldwide remain either unvaccinated or dropped out, which is even worse in sub-Saharan Africa (26.06%) [5, 6]. Among the children who remain unvaccinated or drop out, approximately 10 million infants (49%) live in fragile or humanitarian settings, including countries impacted by conflict. Children, who reside in such challenging circumstances, are the most vulnerable to disease outbreaks and require urgent attention and support [7].

The Expanded Program for Immunization (EPI) in Ethiopia recommends that children receive all of the following vaccines within two years of age: one dose of BCG(Bacille-Calmette–Guerin) and oral polio at birth or as soon as possible; three doses of oral polio vaccine (OPV), three doses of pentavalent (Diphtheria, pertussis, tetanus, hepatitis B, and Hib); three doses of pneumococcal vaccines; two doses of rotavirus, and inactivated polio vaccine (IPV) at intervals of four weeks; and measles vaccinations at or soon after the age of 9 months to reduce child morbidity and mortality [8, 9].

The Ethiopian Federal Ministry of Health has organized health extension workers and health facilities to deliver immunization services at static, outreach, and mobile locations to achieve the EPI recommendation. However, according to the Ethiopia Mini Demographic and Health Survey 2019 (EDHS, 2019), only 44% of children aged 12–23 months have received all basic vaccinations, and nearly 56% of children aged from 12 to 22 months have incomplete vaccination or have dropped out [10]. Furthermore, there is significant geographical variation in Ethiopia in terms of vaccination coverage, with the highest percentages in Addis Ababa (83%) and Tigray (73%) and the lowest percentages in Somali (19%) and Afar (20%) [10].

Vaccination dropout indicates immunization coverage, program performance, program continuity, and follow-up. It is determined by calculating the proportion of children who dropped out from the immunization between penta-1 and penta-3. Alternatively, it can also be determined by calculating the proportion of children who did not receive measles vaccination among those who received BCG [11, 12].

Vaccination dropout significantly impacts the health of children, the health system, and the broader community. For children, incomplete immunization increases susceptibility to preventable diseases, leading to higher morbidity and mortality rates. This places a considerable strain on the health system by escalating the demand for treatment of preventable illnesses, diverting resources from other critical health services, and increasing overall healthcare costs. Moreover, vaccination dropout undermines herd immunity, heightening the risk of disease outbreaks and endangering vulnerable populations, including those who cannot be vaccinated for medical reasons. Consequently, addressing vaccination dropout is essential to safeguarding individual and public health, ensuring efficient health system functionality, and fostering community resilience against infectious diseases [1, 2, 4].

The magnitude of vaccination dropout in Ethiopia ranged from 8.6% in the Amhara region [13] to 48% in the Afar region [14] based on BCG to measles. For Penta-1 to Penta-3, the vaccination dropout rate ranges from 50.4% in the Somalia region [15], indicating an inconsistency in vaccination dropout rates among different geographical regions of Ethiopia. Various studies on the vaccination dropout rate in Ethiopia have indicated that socioeconomic and demographic factors, topographical challenges, armed conflict, and maternal knowledge are determinants of vaccination dropout [15–19].

Considering Ethiopia’s political instability and ongoing internal conflict, there is no national aggregated data available, despite numerous primary research studies that have reported the percentage of vaccine dropout rates and the factors associated with them. Therefore, this systematic review and meta-analysis aimed to determine the pooled magnitude of vaccination dropout and associated factors among children aged 12–23 months in Ethiopia. The findings might assist health professionals and other stakeholders in addressing gaps by prioritizing and customizing immunization campaigns and operational plans based on the study’s results.

## Methods and materials

### Study protocol and registration

This systematic review and meta-analysis was conducted to determine vaccination dropout rates among children in Ethiopia according to the requirements of the Preferred Reporting Items for Systematic Reviews and Meta-Analyses (PRISMA) guidelines [20]. We tracked the flowchart from the PRISMA guidelines to show the selection process. This study was registered in the International Prospective Register of Systematic Reviews (PROSPERO) database with protocol number, CRD42024526789.

### Search strategy

A comprehensive search was conducted using various international online databases, including PubMed, ScienceDirect, Scopus, Cochrane, Web of Science, and Google, as well as repositories from Ethiopian universities, covering studies published between 2014 and 2024. This search aimed to identify articles on the magnitude of vaccination dropout in Ethiopia, following the PRISMA statement [20]. Two members of the review team (EBG and HG) searched the database independently from February 15 to March 6, 2024. The following key search terms were used to search articles: ((((Dropout) OR (“Dropout” OR “Failure to complete”)) AND Immunization) OR (“Immunization” OR “Vaccine” OR “Vaccination”)) AND Children) OR (“Child” OR “Childhood” OR “Baby”) AND Ethiopia.

### Eligibility criteria

The eligibility criteria for this review were based on condition, context, and population mnemonic (CoCoPop) questions for prevalence studies. Correspondingly, published studies conducted only in Ethiopia and reporting the magnitude of the vaccine dropout rate among children aged 12–23 months, articles that were published in the English language, and fulfilled the following criteria (Table 1) were included in this review.

**Table 1:** Inclusion and exclusion criteria

Study characteristics	Inclusion criteria	Exclusion criteria
Design	Cross sectional studies	Other than cross sectional studies
Population	children aged 12–23 months	Children older than 2 Years
Condition	Vaccination dropout rate	Children who did not start their vaccination
Context	Studies conducted in Ethiopia	Studies conducted other than Ethiopia
Language	Published in English	Published in English
Time period of publication	From Jan, 2014 GC to Sep, 2024	Published articles before 2014

### Outcome measurement

The outcome variable of the current study was vaccination dropout, which was the proportion of children who dropped out from immunization between penta-1 and penta-3 or who did not receive measles (MCV 1) vaccination among those who received BCG [14].

### Data extraction

Data were extracted from the included studies via a standardized data extraction Microsoft Excel spreadsheet. The spreadsheet included the first author’s name, publication year, study period, study design, study area, sample size, response rate, and magnitude of vaccine dropout. (Additional file 1).

### Quality assessment of the studies

The Joanna Briggs Institute (JBI) critical appraisal checklist for cross-sectional studies was used to assess the quality of the included studies [21]. Three authors (SG, MAS, MA, and HK) independently appraised the standards of the included studies. The critical analysis checklist has eight parameters; yes, no, unclear, and not applicable options. Then a composite quality index was grouped and the risk of bias was ranked as low risk when it was 50% or above on the quality assessment indicators. Finally, articles with low risks of bias were considered for this systematic review and meta-analysis (Additional file 2).

### Statistical methods and analysis

After all relevant findings were extracted via a Microsoft Excel spreadsheet; the data were exported to STATA-17 software for analysis. The standard error (SE) of the magnitude of the vaccination dropout rate was determined. The I^2^ statistical test with the corresponding p-value was computed to check heterogeneity across the studies [22]. As a result of significant heterogeneity, a random-effects model using the DerSimonian-Laird method corresponding to the 95% CI was used to estimate the pooled magnitude of vaccination dropout [23]. The publication bias was examined using both qualitatively via funnel plots and quantitatively via Egger’s regression test [24]. A p-value less than 0.05 was used to declare the presence of publication bias. Sensitivity analysis was also performed to assess the effect of a single study on the overall magnitude of the meta-analysis estimate. The findings of the study were then presented in the form of text, tables, and figures.

## Results and discussion

### Search results and study selection

**Fig. 1:**
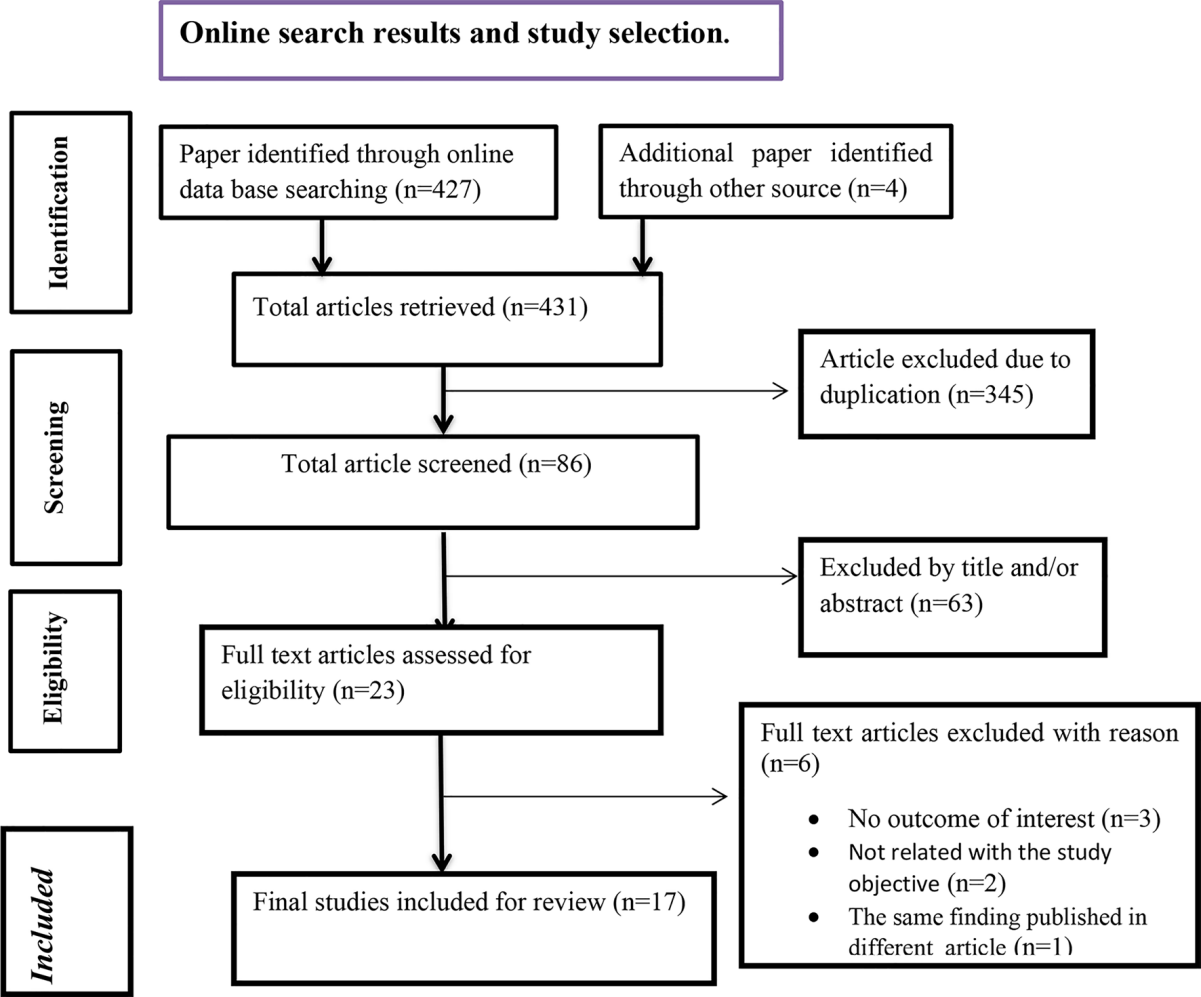
PRISMA flow diagram describing the selection of studies in the systematic review and meta-analysis for the magnitude of vaccination dropout among children in Ethiopia

A search for studies using online search engines such as PubMed, Scopus, Google Scholar, Science Direct, and the institutional repositories of Ethiopian universities retrieved a total of 431 published and unpublished studies. After excluding 408 studies based on title and abstract screening, 23 full-text papers were obtained. Ultimately, 17 articles [13–15, 25–38], involving 9,152 study participants, met the inclusion criteria and were included in this systematic review and meta-analysis (Fig. 1).

### Characteristics of the selected studies

Eight studies were conducted in the Amhara region [13, 25–29, 35, 36], three in the Oromia region [30, 32, 38], two in the SNNPR region [33, 34], and one each in Gambela [31], Afar [14], Harar [37], and Somalia [15]. All the studies employed a cross-sectional study design. Regarding vaccine dropout types, eleven studies reported dropout rates for both Penta-1 to Penta-3 and BCG to measles [13–15, 25, 27–30, 32, 33, 35], five studies reported dropout rates from BCG to measles only [26, 31, 34, 36, 37], and one study reported the dropout rate from Penta-1 to Penta-3 only [38]. A total of sixteen and twelve studies were included to determine the pooled magnitude of BCG to measles dropout and Penta-1 to Penta-3 dropout, respectively (Table 2).

**Table 2:** Characteristics of the studies included in this systematic review and meta-Analysis

Author/Year	Study region	Sample size	Vaccine dropout from	Prevalence %	Quality assessment
Mekonnen et al.,2019 [25]	Amhara	566	BCG to measles	9.17	Low risk
			Penta1-Penta3	9.07	
Dessalegn et al.,2019 [26]	Amhara	621	BCG to measles	8.6	Low risk
Girmay et al.,2019 [27]	Amhara	620	BCG to measles	10.7	Low risk
			Penta1-Penta3	13.4	
Abebe et al.,2019 [28]	Amhara	389	BCG to measles	9.0	Low risk
			Penta1-Penta3	2.4	
Legesse et al.,2022 [29]	Amhara	591	BCG to measles	15.8	Low risk
			Penta1-Penta3	11.8	
Negero et al.,2019 [30]	Oromia	436	BCG to measles	16.1	Low risk
			Penta1-Penta3	11.8	
Kassahun et al.,2015 [31]	Amhara	751	BCG to measles	6.5	Low risk
			Penta1-Penta3	2.7	
Kebede et al.,2021 [13]	Gambela	422	BCG to measles	25.8	Low risk
Mebrate et al.,2022 [32]	Oromia	657	BCG to measles	22.0	Low risk
			Penta1-Penta3	0.97	
Facha et al., 2015 [33]	SNNPR	210	BCG to measles	11.7	Low risk
			Penta1-Penta3	7.3	
Tarekegn et al.,2018 [14]	Afar	408	BCG to measles	48.0	Low risk
			Penta1-Penta3	30.0	
Beyene et al.,2016 [34]	SNNPR	374	BCG to measles	24.1	Low risk
Tesfaye et al.,2019 [35]	Amhara	830	BCG to measles	7.44	Low risk
			Penta1-Penta3	3.74	
Yehualshet et al.,2019 [36]	Amhara	392	BCG to measles	9.3	Low risk
Yilma et al.,2021 [37]	Harar	409	BCG to measles	9.0	Low risk
Yadita et al.,2021 [15]	Somalia	602	BCG to measles	40.4	Low risk
			Penta1-Penta3	50.4	
Muluye et al.,2022 [38]	Oromia	874	Penta1-Penta3	17.0	Low risk

BCG: Bacillus Calmette–Guérin SNNPR: Southern Nations and Nationalities of People Region

### The magnitude of vaccine dropout among children in Ethiopia

**Fig. 2:**
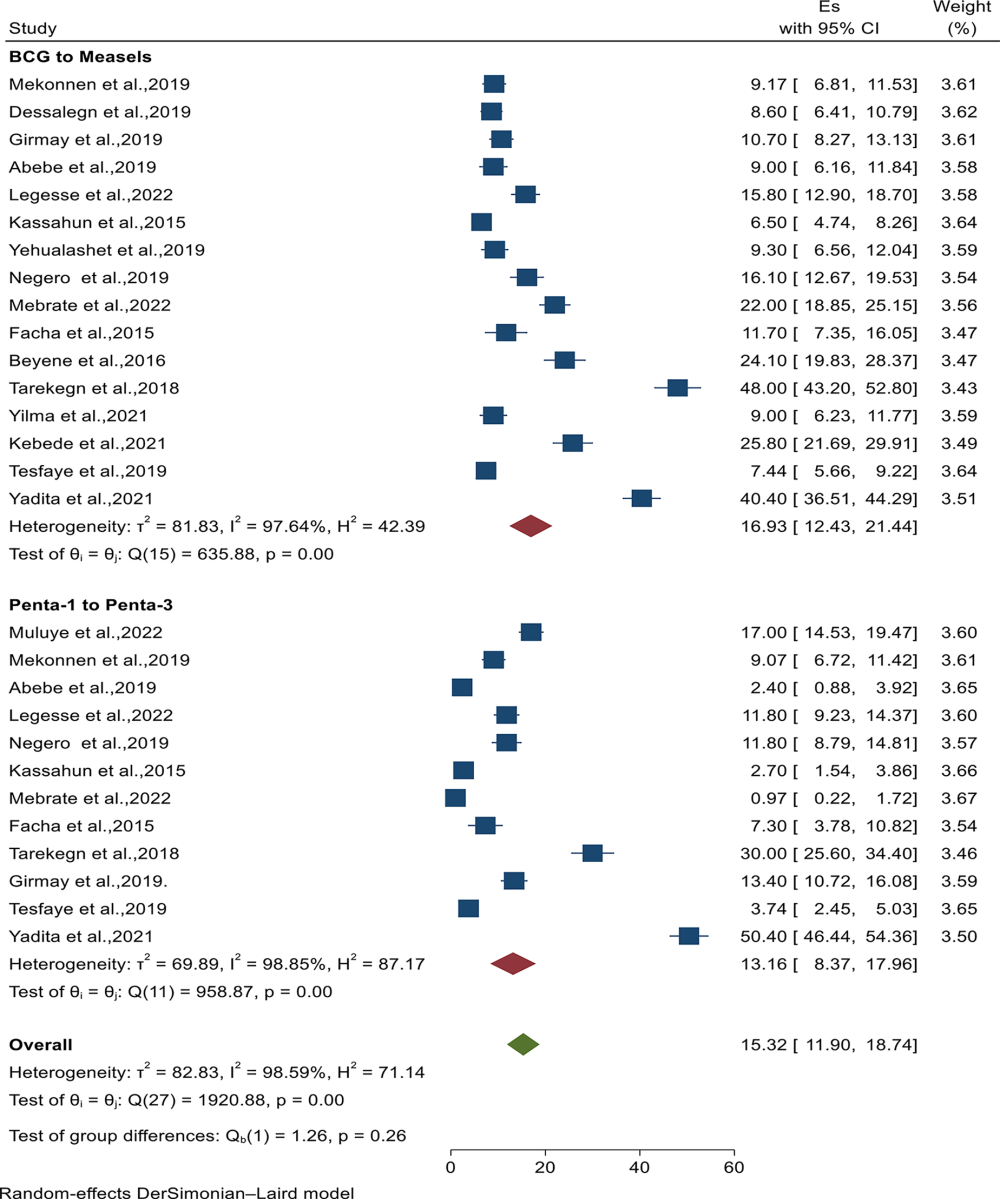
Forest plot diagram indicating the pooled magnitude of vaccine dropout rate among children in Ethiopia

The pooled magnitude of vaccination dropout from BCG to measles among children aged 12–23 months in Ethiopia from a random-effects model was 16.93% (95% CI: 12.43, 21.44) with a heterogeneity index (*I*^*2*^) of 97.64%. Similarly, the pooled magnitude of Penta-1 to Penta-3 dropout was 13.16% (95% CI: 8.37, 17.96) with a heterogeneity index (*I*^*2*^) of 98.85% (Fig. 2).

### Heterogeneity and sensitivity analysis

**Fig. 3:**
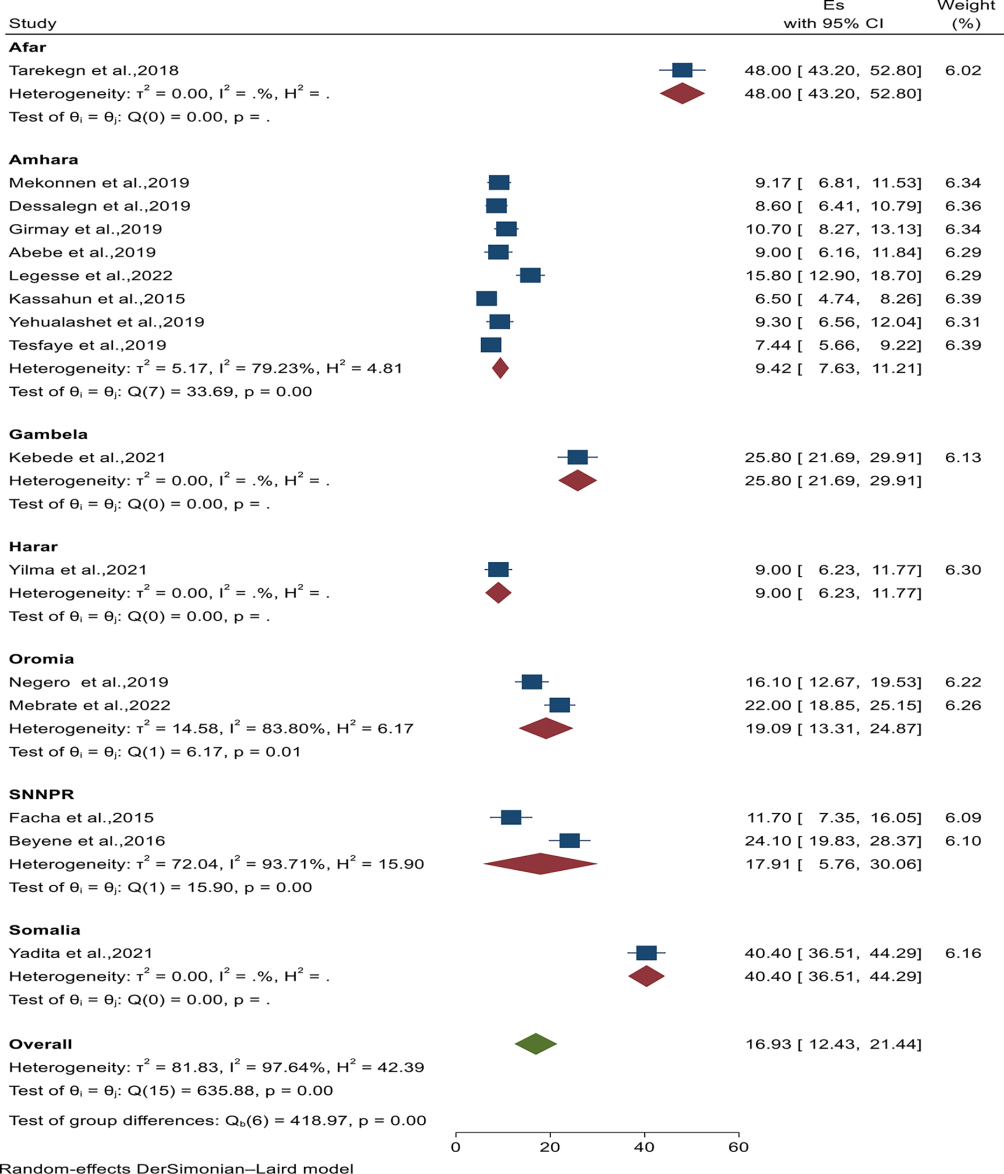
Forest plot displaying subgroup analysis from BCG to measles based on region

**Fig. 4:**
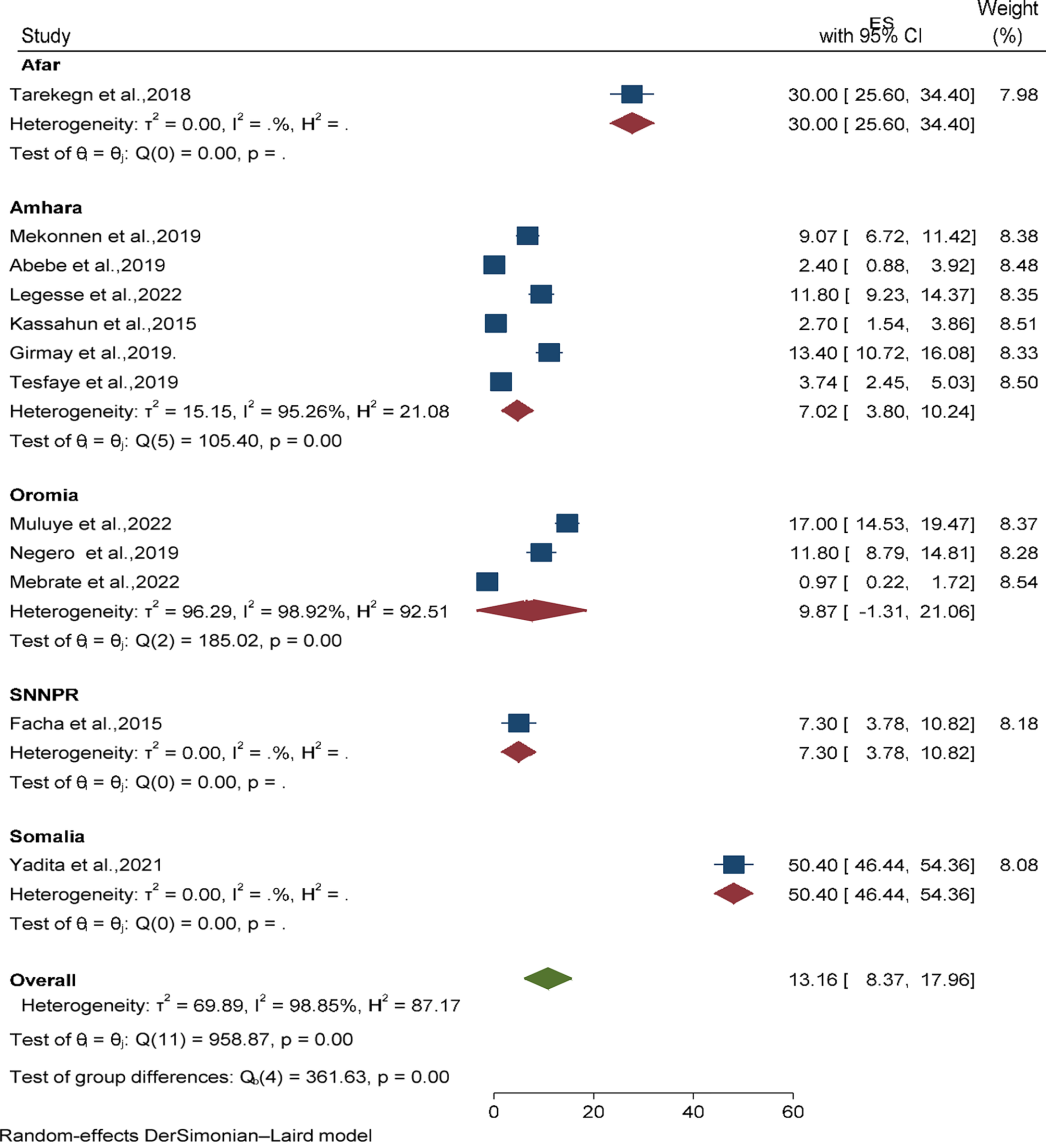
Forest plot displaying subgroup analysis of Penta1- to Penta-3 vaccine dropout among children in Ethiopia by region

A substantial level of heterogeneity (*I*^*2*^ = 98%) was observed between included studies; for that to explore the between-study heterogeneity, sub-group analysis was conducted by the region where the studies were conducted, and there was a statistically significant difference in the magnitude of vaccine dropout rate in the region. Accordingly, the Harar region had the lowest magnitude of BCG to measles dropout (9.0%; 95% CI: 6.23, 11.77), whereas the Afar region had the highest dropout (48%; 95% CI: 43.2, 58.2) (Fig. 3). On the other hand, the Amhara region had the lowest magnitude of Penta-1 to Penta-3 dropout (7.02%; 95% CI: 3.80, 10.24; I^2^ = 95.26%), whereas the Somalia region had the highest (50.4%; 95% CI: 46.44, 54.36) (Fig. 4).

Subgroup analysis was also done based on sample size (< 500 and *≥* 500), and publication year ( before 2019 and after 2019), yet, no significant variation was detected. Besides, the random effect meta-regression was performed to further explore the source of heterogeneity by considering sample size and publication year as covariates. Consequently, the results indicated that sample size (*p* =.761) and publication year (*p* =.639) did not affect heterogeneity between studies.

The leave-one-out sensitivity analysis was also performed to examine any outliers in the overall estimate of the pooled magnitude. As a result, studies omitted at a time did not show a significant change in the overall magnitude of vaccination dropout. Hence, the point estimate of vaccine dropout rate for each omitted analysis lies within the CI of the combined analysis (Tables 3 and 4).

**Table 3:**
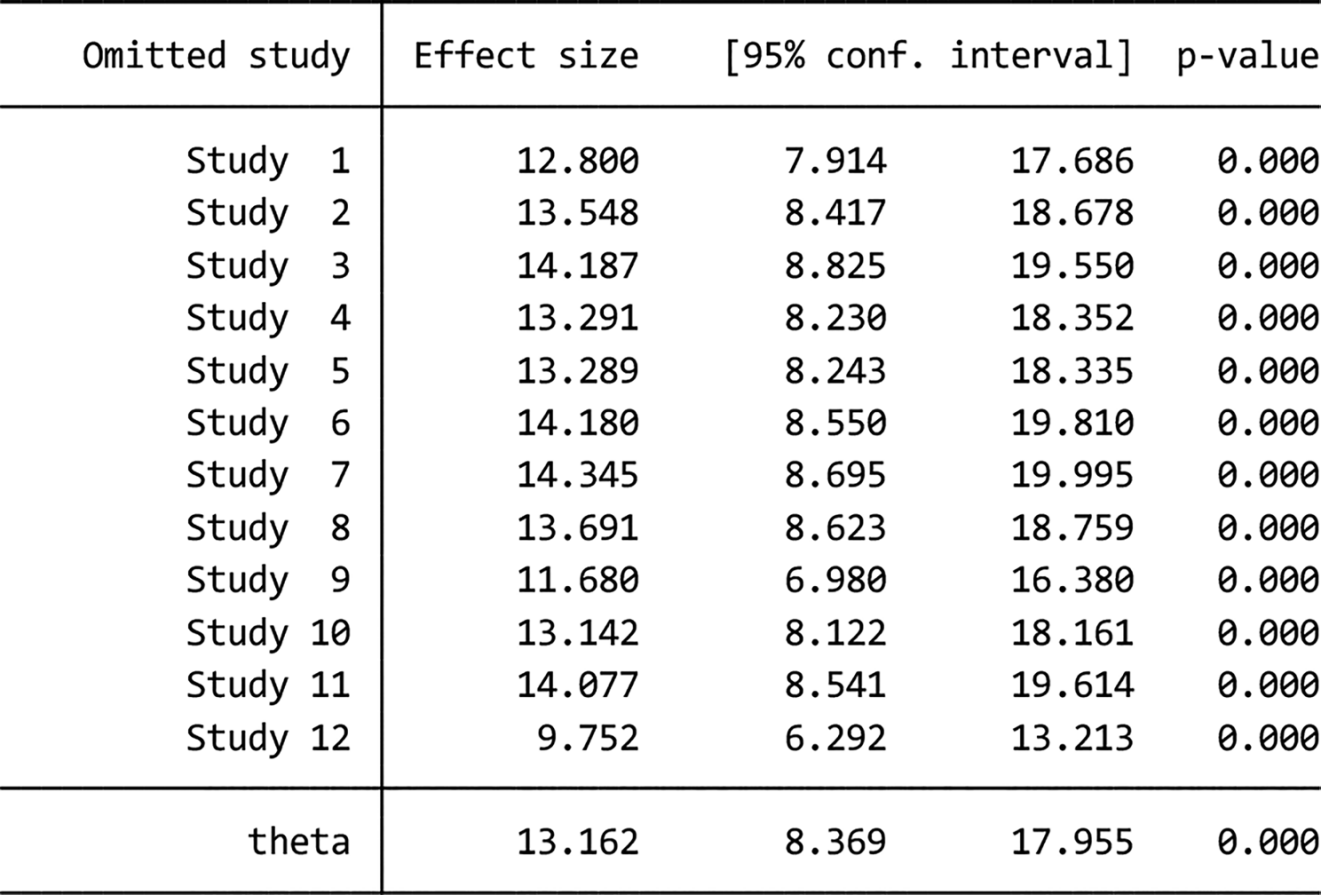
Sensitivity analysis for the effect of each study on the summary estimate (Pent-1 to penta-3 dropout)

**Table 4:**
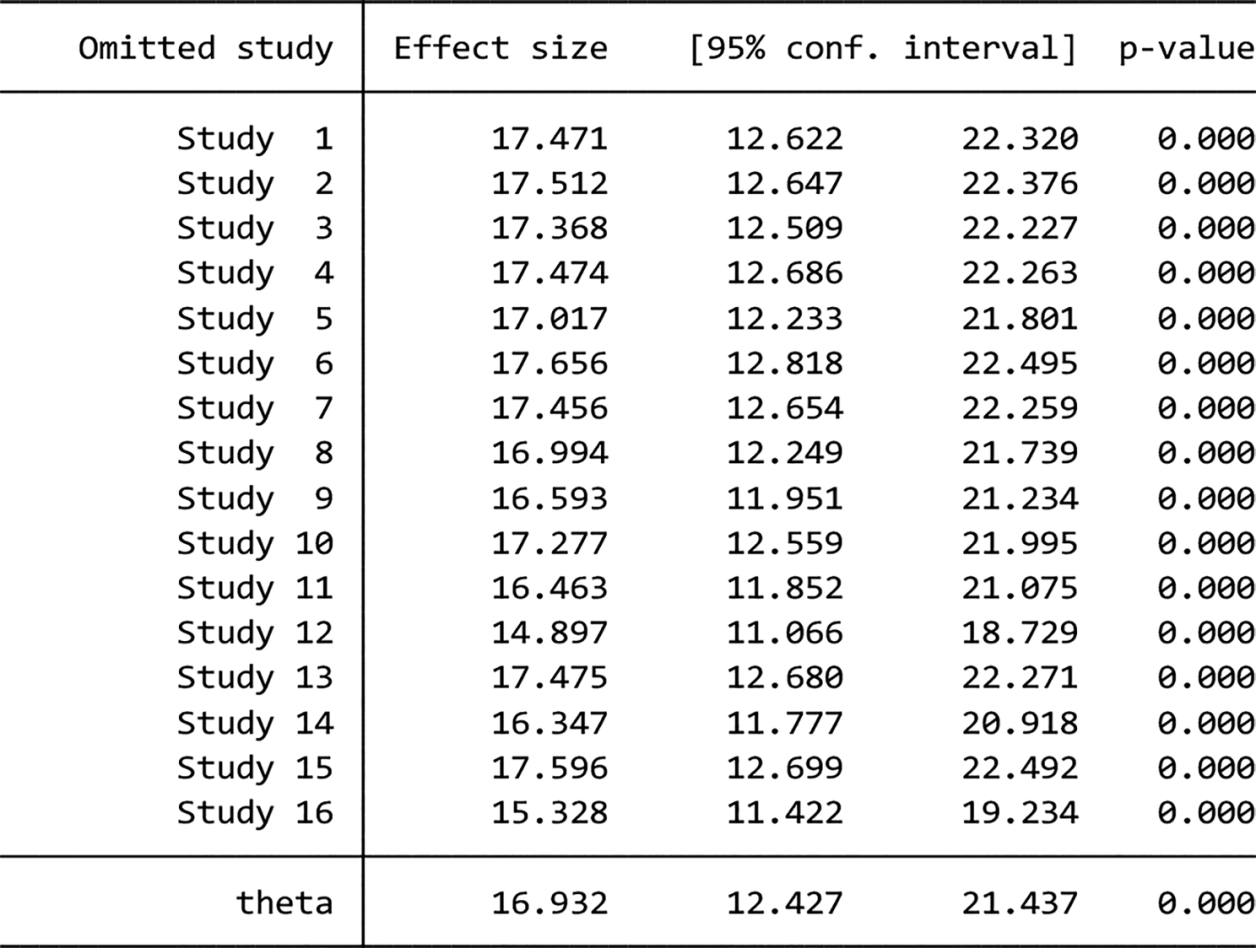
Sensitivity analysis for the effect of each study on the summary estimate (BCG to measles dropout)

### Publication bias

**Fig. 5:**
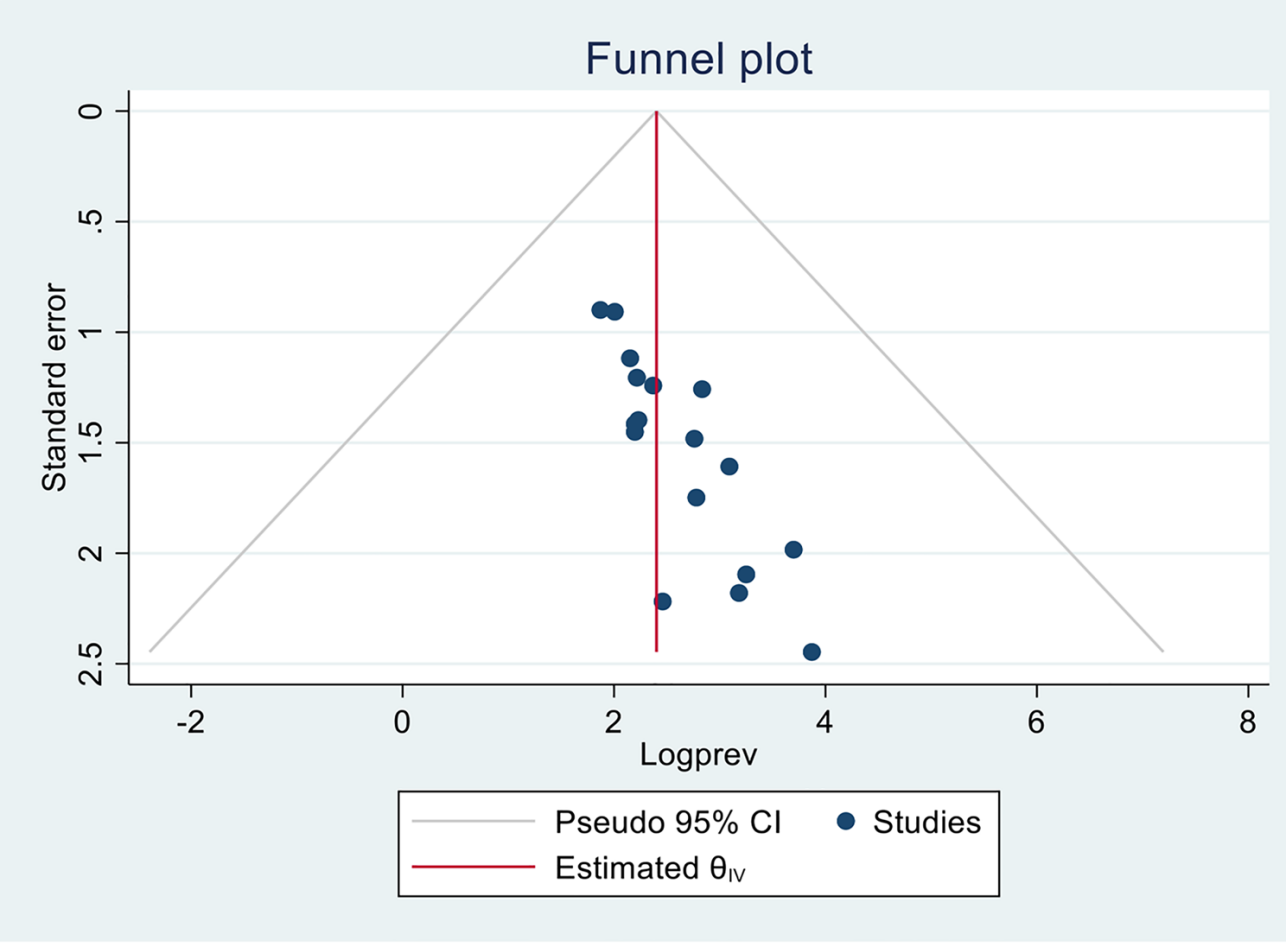
Funnel plot displaying a symmetrical distribution of the included studies

The likelihood of publication bias across studies was evaluated via funnel plot and Egger’s regression test. The symmetrical funnel plot (Fig. 5) clearly illustrates that there was no indication of publication bias in the included studies. Additionally, the results of Egger’s test (*p* =.22), confirmed this finding.

### Determinants of vaccination dropout among children in Ethiopia

In this meta-analysis, the pooled effects of factors associated with vaccine dropout among children were estimated. Accordingly, four variables (not attending ANC, postponed immunization schedule, distance from the health care facility and home delivery) were identified as significant factors associated with vaccine dropout. The pooled effects of four studies [26, 31, 34, 38] showed that mothers/caregivers who did not attend ANC were 3.58 times more likely to have children who dropped out of vaccination compared to those who attended ANC (AOR = 3.58, 95% CI: 1.99–6.44). Additionally, three studies [31, 34, 36] assessed the pooled odds ratio for the association between a postponed immunization schedule and vaccine dropout. Accordingly, those with a postponed immunization schedule were 2.73 times more likely to drop out from vaccine compared to the counterparts (AOR = 2.73, 95% CI = 1.53, 4.87). Furthermore, in this meta-analysis, the pooled effects of distance from health care facilities on vaccine dropout were assessed using the findings of five studies [25, 26, 31, 34, 38]. The results indicated that women who traveled more than 30 min to reach a health institution were 2.46 times more likely to experience vaccination dropout compared to their counterparts (AOR = 2.46, 95% CI: 2.01–17.18). Additionally, the pooled analysis of two studies [30, 31] revealed that women who delivered at home were 2.78 times more likely to drop out of vaccination compared to those who delivered at a health institution (AOR = 2.78, 95% CI: 2.28–3.38) (Table 5).

**Table 5:** Summary estimate of ORs for vaccine dropout associated factors

Variables	Included studies	OR(95%CI)	I^2^
Not Attended ANC	4 [26, 31, 34, 38]	3.58(1.99–6.44)	24.7%
Postponed immunization schedule	3[31, 34, 36]	2.73(1.53–4.87	17.1%
Distance from the Health facility > 30 min	5[25, 26, 31, 34, 38]	2.46(2.01–17.18)	44.2%
Home delivery	3[30, 31, 30]	2.78(2.28–3.38)	11.3%)

## Discussion

Children in sub-Saharan Africa, particularly in Ethiopia, continue to suffer from vaccine-preventable diseases (VPDs), contributing to child mortality. One of the key challenges is vaccination dropout, where children fail to receive subsequent doses after the initial vaccine, leading to incomplete immunization coverage [39].

Our meta-analysis revealed that the pooled magnitude of vaccination dropout for BCG-to-measles among children aged 12–23 months in Ethiopia was found to be 16.93% (95% CI: 12.43, 21.44). This finding was consistent with the EDHS 2019 data (18%) [10], studies in Mozambique (19%) [40], and those in Iraq (19.3%) [41]. This finding is higher than those of studies in Gambia (6.8%) [42] and India (8.6%) [43]. However, our findings were lower than the studies conducted in Ghana (31.5%) and Cameron (50%) [16, 44]. A possible explanation might be differences in the study setting and population.

Similarly, the overall pooled magnitude of vaccination dropout for Penta-1-to-Penta-3 was found to be 13.16% (95% CI: 8.37, 17.96). This finding is consistent with the EDHS 2019 data (15%) [10], studies in Kenya (13.9%) [6], and Ghana (11.13) [6]. The present finding is higher than studies in Gambia (4.3%) [42], and India (7.4%) [43]. However, our finding is lower than the previous meta-analysis conducted in sub-Saharan Africa (21.06%) [6], and Nepal (28.35%) [45]. These variations may be influenced by differences in health system structures, service delivery, and healthcare accessibility across countries. For instance, nations with robust immunization tracking systems, and integrated primary healthcare networks may exhibit lower dropout rates, as they ensure timely follow-ups and community-based interventions.

Our meta-analysis revealed that the pooled magnitude of vaccination dropout in Ethiopia exceeds the WHO-recommended threshold of less than 10%, with dropout rates of 16.93% for BCG-to-measles and 13.16% for Penta-1-to-Penta-3 [46]. The WHO emphasizes that if the dropout rate between Penta-1 and Penta-3, or between BCG as the entry vaccine and measles as the exit vaccine, exceeds 10%, it indicates an issue with either access or service utilization. These may stem from differences in health infrastructure, and the efficiency of immunization programs across various settings. Therefore, the analysis highlights not only gaps in Ethiopia’s immunization program but also underscores the importance of strengthening healthcare systems to improve vaccination dropout.

In this review, subgroup analysis revealed that the Afar region (48%) and the Somalia region (50.4%) had the highest magnitudes of BCG-to-measles and Penta-1-to-Penta-3 dropouts, respectively. This finding aligns with other studies conducted in Ethiopia [17, 47]. The predominantly pastoralist and nomadic lifestyle of the populations in these regions likely contributes to this issue, as families frequently move in search of grazing grounds, often without long-term planning. This lifestyle hinders children from accessing healthcare services, including immunizations. Therefore, these regions may require targeted strategies, such as creating a network of informants and influencers and implementing vaccination campaigns, to reduce dropout rates [48].

This review also identified determinants of vaccine dropout among children. Not attending ANC, postponing immunization schedules, distance from the healthcare facility, and home delivery were identified as significant factors associated with vaccine dropout. Mothers/caretakers who did not attend ANC were about 3.58 times more likely to have a dropout from vaccination compared to women who attended ANC follow-up. This result is consistent with studies conducted in Nigeria [49], India [43], and Nepal [50]. This may be because mothers without ANC follow-up miss out on critical information and reminders about immunization, which are often provided through repeated education during ANC visits. Antenatal follow-up serves as an opportunity to educate mothers and caregivers on the benefits of vaccination. Similarly, studies have demonstrated that mothers who attend ANC are significantly more likely to fully vaccinate their children. For instance, a meta-analysis reported that mothers with at least one ANC visit were 3.2 times more likely to complete their children’s vaccination schedules. These findings suggest that integrating immunization counseling into ANC services could be an effective strategy to improve vaccination rates [51].

In line with studies conducted in Nigeria [52], Congo [53], and Nepal [50], this review found that women who travel more than 30 min to reach health institutions were more likely to experience vaccination dropout. This may be because rural women in developing nations such as Ethiopia are often responsible for childcare, cooking, and cleaning, leaving them unable to travel long distances, which results in missed vaccinations for their children. A peer-reviewed study also identified long distances to health facilities as a contributing factor to lack of vaccination [54]. However, according to our analysis, distance from the health facility (> 30 min) has a notably wide confidence interval (OR: 2.46, 95% CI: 2.01–17.18), which suggests substantial variability in the findings across the included studies. This variability could be due to differences in study populations, geographic locations, or health system characteristics that might influence the impact of distance on health outcomes.

Additionally, the review revealed that children delivered at home were more likely to drop out of vaccination. This finding is consistent with studies conducted in Kenya [55], Nigeria [56], and Pakistan [57]. This may be attributed to healthcare providers raising awareness about the importance of vaccination through counseling and education, which is often missed during home deliveries.

The findings of this systemic review have significant implications for public health policies, strategies, and interventions. It underscores the need for a multifaceted approach to reducing vaccination dropout rates, and improving maternal health engagement, accessibility, and public awareness. These steps can contribute to higher immunization coverage, ultimately improving child health outcomes and reducing preventable diseases in Ethiopia.

### Limitations of the study

This systematic review is subject to certain limitations. One key constraint is the inclusion of only English-language studies, which may introduce language bias. Although efforts were made to identify relevant research, articles published in other languages were excluded due to feasibility constraints and the absence of translation resources. Additionally, while several studies have been conducted within Ethiopia, our analysis incorporates international literature to provide broader comparative insights. However, these comparisons should be interpreted with caution, as healthcare system structures, socioeconomic conditions, and access to services differ significantly between developed and developing regions. Another limitation of this review is the categorization of ‘mothers/caregivers’ as a single group. Since not all caregivers are biological mothers, some may not have attended antenatal care (ANC), potentially skewing the assessment of the association between ANC attendance and vaccination dropout rates.

## Conclusion

This meta-analysis revealed that the pooled magnitude of vaccination dropout among children in Ethiopia exceeds the WHO recommendation of less than 10%. Factors including lack of antenatal care (ANC) attendance, postponement of immunization schedules, geographic barriers to healthcare facilities, and home deliveries, were found to contribute significantly to vaccine dropout.

To address these challenges, targeted interventions are necessary. First, strengthening ANC education and counseling services can improve maternal awareness and encourage timely vaccination. Expanding community-based health worker programs, particularly in rural and underserved regions, can ensure consistent follow-ups and immunization reminders for families. Additionally, implementing mobile clinics and outreach immunization services in hard-to-reach areas can help bridge accessibility gaps. Improving institutional delivery rates is also crucial, as healthcare providers can administer vaccinations and provide immediate postnatal education. Policies supporting free or subsidized maternal healthcare, alongside transportation assistance programs for expectant mothers, may encourage institutional deliveries. These strategies, combined with ongoing health education campaigns and local engagement efforts, will be instrumental in reducing vaccination dropout rates and strengthening Ethiopia’s immunization program.

## Electronic supplementary material

Below is the link to the electronic supplementary material.

Supplementary Material 1: Additional file 1: Extracted data with name of extractors and date of extraction

Supplementary Material 2: Additional file 2: Risk of bias assessment for included studies

Supplementary Material 3: Additional file 3: Excluded studies

Supplementary Material 4: Checklist: PRISMA 2020 Checklist

## Data Availability

All relevant data generated or analysed during this study are included in this published article and its supplementary information files.

## Abbreviations

ANC: Antenatal care
BCG: Bacillus Calmette–Guérin
JBI: Joanna Briggs Institute
PRISMA: Preferred Reporting Items for Systematic Reviews and Meta-Analyses
