# Supplementary material for: Vaccination dropout and associated factors among children in Ethiopia: a systematic review and meta-analysis (2014–2024)
Source: BMC Pediatr. 2025 May 28;25:426. doi: 10.1186/s12887-025-05786-3 (PMC12117780; doi:10.1186/s12887-025-05786-3)
Supplement: Supplementary file 1 — Supplementary Material 1: Additional file 1: Extracted data with name of extractors and date of extraction [file 12887_2025_5786_MOESM1_ESM.docx]

Additional file 1: Extracted data with name of extractors and date of extraction

| **Author/Year** | **Study design** | **Sample size** | **Vaccine dropout**  **from** | **Prevalence %** | **Date of data extraction** | **Data extractors** | **Eligibility for inclusion** |
| --- | --- | --- | --- | --- | --- | --- | --- |
| Mekonnen et al,2019 [25] | Cross-sectional | 566 | BCG to measles | 9.17 | 28/07/2024 G.C | Eyasu Bamlaku and Habtamu Geremew | Eligible |
|  |  |  | Penta1-Penta3 | 9.07 |  |  |  |
| Dessalegn et al.,2019 [26] | Cross-sectional | 621 | BCG to measles | 8.6 | 30/07/2024 G.C | Eyasu Bamlaku and Alegntaw Abate | Eligible |
| Girmay et al.,2019 [27] | Cross-sectional | 620 | BCG to measles | 10.7 | 04/08/2024 G.C | Eyasu Bamlaku and Mulat Belay Simegn | Eligible |
|  |  |  | Penta1-Penta3 | 13.4 |  |  |  |
| Abebe et al.,2019 [28] | Cross-sectional | 389 | BCG to measles | 9.0 | 04/08/2024 G.C | Eyasu Bamlaku and Mohammed Ahmed Ali | Eligible |
|  |  |  | Penta1-Penta3 | 2.4 |  |  |  |
| Legesse et al.,2022 [29] | Cross-sectional | 591 | BCG to measles | 15.8 | 28/08/2024 G.C | Eyasu Bamlaku and Werkneh Melkie | Eligible |
|  |  |  | Penta1-Penta3 | 11.8 |  |  |  |
| Negero et al.,2019 [30] | Cross-sectional | 436 | BCG to measles | 16.1 | 02/08/2024 G.C | Eyasu Bamlaku and Samuel Abdisa | Eligible |
|  |  |  | Penta1-Penta3 | 11.8 |  |  |  |
| Kassahun et al.,2015 [31] | Cross-sectional | 751 | BCG to measles | 6.5 | 06/08/2024 G.C | Eyasu Bamlaku and Meron Admasu | Eligible |
|  |  |  | Penta1-Penta3 | 2.7 |  |  |  |
| Kebede et al.,2021 [13] | Cross-sectional | 422 | BCG to measles | 25.8 | 06/08/2024 G.C | Eyasu Bamlaku and Meron Admasu | Eligible |
| Mebrate et al.,2022 [32] | Cross-sectional | 657 | BCG to measles | 22.0 | 30/08/2024 G.C | Eyasu Bamlaku and Smegnew Gichew | Eligible |
|  |  |  | Penta1-Penta3 | 0.97 |  |  |  |
| Facha et al. ,2015[33] | Cross-sectional | 210 | BCG to measles | 11.7 | 02/08/2024 G.C | Eyasu Bamlaku and Samuel Abdisa | Eligible |
|  |  |  | Penta1-Penta3 | 7.3 |  |  |  |
| Tarekegn et al.,2018[14] | Cross-sectional | 408 | BCG to measles | 48.0 | 28/08/2024 G.C | Eyasu Bamlaku and Habtamu Geremew | Eligible |
|  |  |  | Penta1-Penta3 | 30.0 |  |  |  |
| Beyene et al.,2016[34] | Cross-sectional | 374 | BCG to measles | 24.1 | 04/08/2024 G.C | Eyasu Bamlaku and Mohammed Ahmed Ali | Eligible |
| Tesfaye et al.,2019 [35] | Cross-sectional | 830 | BCG to measles | 7.44 | 04/08/2024 G.C | Eyasu Bamlaku and Mohammed Ahmed Ali | Eligible |
|  |  |  | Penta1-Penta3 | 3.74 |  |  |  |
| Yehualshet et al.,2019 [36] | Cross-sectional | 392 | BCG to measles | 9.3 | 06/08/2024 G.C | Eyasu Bamlaku and Alegntaw Abate | Eligible |
| Yilma et al.,2021 [37] | Cross-sectional | 409 | BCG to measles | 9.0 | 29/07/2024 G.C | Eyasu Bamlaku and Habtamu Geremew | Eligible |
| Yadita et al.,2021 [15] | Cross-sectional | 602 | BCG to measles | 40.4 | 01/08/2024 G.C | Eyasu Bamlaku and Alegntaw Abate | Eligible |
|  |  |  | Penta1-Penta3 | 50.4 |  |  |  |
| Muluye et al.,2022 [38] | Cross-sectional | 874 | Penta1-Penta3 | 17.0 | 06/08/2024 G.C | Eyasu Bamlaku and Meron Admasu | Eligible |

*BCG: Bacillus Calmette–Guérin*
