# Supplementary material for: Vaccination dropout and associated factors among children in Ethiopia: a systematic review and meta-analysis (2014–2024)
Source: BMC Pediatr. 2025 May 28;25:426. doi: 10.1186/s12887-025-05786-3 (PMC12117780; doi:10.1186/s12887-025-05786-3)
Supplement: Supplementary file 2 — Supplementary Material 2: Additional file 2: Risk of bias assessment for included studies [file 12887_2025_5786_MOESM2_ESM.docx]

SP File 3: Studies excluded after full text review

| **No** | **Study** | **Reason for exclusion** |
| --- | --- | --- |
| 1 | Shiferie F, Gebremedhin S, Andargie G, Tsegaye DA, Alemayehu WA, Mekuria LA, Wondie T, Fenta TG. Vaccination dropout and wealth related inequality among children aged 12-35 months in remote and underserved settings of Ethiopia: a cross-sectional evaluation survey. Front Pediatr. 2023 Oct 24;11:1280746. doi: 10.3389/fped.2023.1280746. PMID: 37941975; PMCID: PMC10628708 | Outcome of interest (Population difference) |
| 2 | Nigatu T, Abraham L, Willems H, et al. The status of immunization program and challenges in Ethiopia: A mixed method study. *SAGE Open Medicine*. 2024;12. doi:[10.1177/20503121241237115](https://doi.org/10.1177/20503121241237115) | Have no outcome of interest |
| 3 | Tesfaye TD, Temesgen WA, Kasa AS. Vaccination coverage and associated factors among children aged 12 - 23 months in Northwest Ethiopia. Hum Vaccin Immunother. 2018;14(10):2348-2354. doi: 10.1080/21645515.2018.1502528. Epub 2018 Sep 5. PMID: 30118398; PMCID: PMC6284506 | Not related with the study objective |
| 4 | Tolera, M., Birhanu, A., Regassa, L.D. *et al.* Further analysis of determinants of Pentavalent and Measles immunizations dropouts among children under five years of age in Ethiopia from Mini-EDHS 2019. *BMC Health Serv Res* **24**, 1527 (2024). https://doi.org/10.1186/s12913-024-11573-0 | Outcome of interest (Population difference) |
| 5 | Hailu, C., Fisseha, G. & Gebreyesus, A. Measles vaccination dropout among 12 − 23 months aged children in pastoralist community of Afar, Ethiopia. *BMC Infect Dis* **22**, 376 (2022). https://doi.org/10.1186/s12879-022-07350-1 | Not related with the study objective |
| 6 | Fenta, S.M., Fenta, H.M. Individual and community-level determinants of childhood vaccination in Ethiopia. *Arch Public Health* **79**, 53 (2021). https://doi.org/10.1186/s13690-021-00581-9 | The same finding published in different article |
