# Supplementary material for: Vaccination dropout and associated factors among children in Ethiopia: a systematic review and meta-analysis (2014–2024)
Source: BMC Pediatr. 2025 May 28;25:426. doi: 10.1186/s12887-025-05786-3 (PMC12117780; doi:10.1186/s12887-025-05786-3)
Supplement: Supplementary file 3 — Supplementary Material 3: Additional file 3: Excluded studies [file 12887_2025_5786_MOESM3_ESM.docx]

Additional file 2: Risk of bias assessment for included studies

| **JBI Critical Appraisal Checklist** | | | | | | | | | |  |
| --- | --- | --- | --- | --- | --- | --- | --- | --- | --- | --- |
| **References** | **Criteria for inclusion in the sample clearly defined** | **Study subjects & setting described detail** | **Exposure measure in a valid & reliable way** | **Objective, standard criteria for measure of the event** | **confounding factors identified** | **strategies to deal confounding factors stated** | **Outcome measured in a valid & reliable way** | **Appropriate statistical analysis used** | **score** | **Overall quality** |
| Mekonnen et al.[25] | Yes | Yes | Yes | Yes | No | Yes | Yes | Yes | 7 | Low risk |
| Dessalegn et al.[26] | Yes | Yes | Yes | Yes | No | Yes | Yes | Yes | 7 | Low risk |
| Girmayet al.[27] | Yes | Yes | Yes | Yes | No | Yes | Yes | Yes | 7 | Low risk |
| Abebe et al.[28] | Yes | Yes | Yes | Yes | No | Yes | Yes | Yes | 7 | Low risk |
| Legesse et al.[29] | Yes | Yes | Yes | Yes | No | Yes | Yes | Yes | 7 | Low risk |
| Negero et al.[30] | Yes | Yes | Yes | Yes | No | Yes | Yes | Yes | 7 | Low risk |
| Kassahun et al.[31] | Yes | Yes | Yes | Yes | No | Yes | Yes | Yes | 7 | Low risk |
| Kebede et al.[13] | Yes | Yes | Yes | Yes | No | Yes | Yes | Yes | 7 | Low risk |
| Mebrate et al.[32] | Yes | Yes | Yes | Yes | No | Yes | Yes | Yes | 7 | Low risk |
| Facha et al.[33] | Yes | Yes | Yes | Yes | No | Yes | Yes | Yes | 7 | Low risk |
| Tarekegn et al. [14] | Yes | Yes | Yes | Yes | No | Yes | Yes | Yes | 7 | Low risk |
| Beyene et al. [34] | Yes | Yes | Yes | Yes | No | Yes | Yes | Yes | 7 | Low risk |
| Tesfaye et al.[35] | Yes | Yes | Yes | Yes | No | Yes | Yes | Yes | 7 | Low risk |
| Yehulashet et al.[36] | Yes | Yes | Yes | Yes | No | Yes | Yes | Yes | 7 | Low risk |
| Yilma et al.[37] | Yes | Yes | Yes | Yes | No | Yes | Yes | Yes | 7 | Low risk |
| Yadeta et al.[15] | Yes | Yes | Yes | Yes | No | Yes | Yes | Yes | 7 | Low risk |
| Muluye et al.[38] | Yes | Yes | Yes | Yes | No | Yes | Yes | Yes | 7 | Low risk |
